# Shared Book Reading Promotes Not Only Language Development, But Also Grapheme Awareness in German Kindergarten Children

**DOI:** 10.3389/fpsyg.2017.00364

**Published:** 2017-03-21

**Authors:** Patricia B. C. Wesseling, Corinna A. Christmann, Thomas Lachmann

**Affiliations:** ^1^Cognitive and Developmental Psychology Unit, Center for Cognitive Science, University of Kaiserslautern, KaiserslauternGermany; ^2^Junior Research Group wearHEALTH, Department of Computer Science, University of Kaiserslautern, KaiserslauternGermany

**Keywords:** literacy enrichment, letter knowledge, expressive vocabulary, dialogic reading, reading development, alphabetic phase, letter processing, implicit learning

## Abstract

Effects of shared book reading on expressive vocabulary and grapheme awareness without letter instruction in German kindergarteners (longitudinal; *N* = 69, 3;0–4;8 years) were investigated. Expressive vocabulary was measured by using a standardized test; grapheme awareness was measured by asking children to identify one grapheme per trial presented amongst non-letter distractors. Two methods of shared book reading were investigated, literacy enrichment (additional books) and teacher training in shared book reading strategies, both without explicit letter instruction. Whereas positive effects of shared book reading on expressive vocabulary were evident in numerous previous studies, the impact of shared book reading on grapheme awareness has not yet been investigated. Both methods resulted in positive effects on children’s expressive vocabulary and grapheme awareness over a period of 6 months. Thus, early shared book reading may not only be considered to be a tool for promoting the development of expressive vocabulary, but also for implicit acquisition of grapheme awareness. The latter is considered an important precondition required for the explicit learning of grapheme–phoneme conversion rules (letter knowledge).

## Introduction

Children need intensive and diverse input in order to develop language skills properly (Huttenlocher et al., 1991). Shared book reading with adults represents one important potential to implement such an input (see Sénéchal et al., 1998). For instance, story books contain clues that help one to understand the meaning of unknown words and, therefore, shared book reading provides a tool for the promotion of language development. Positive effects of shared book reading, either with parents or teachers, on children’s receptive and expressive language development, such as growth of vocabulary, and grammar skills, have been reported frequently (e.g., Bus et al., 1995; Neuman, 1996; Sénéchal et al., 1996; Wasik and Bond, 2001; DeTemple and Snow, 2003; Stahl, 2003; Ennemoser et al., 2013).

Aside from its effects on the development of spoken language skills, early shared book reading was also shown to have a positive effect on later literacy development (Brown et al., 1986; Mol and Bus, 2011; Sim and Berthelsen, 2014). In most studies showing this effect, however, an additional explicit instruction of grapheme–phoneme conversion rules was given, i.e., within the context of shared book reading the child was actually taught letter knowledge (Ezell and Justice, 2000; Lovelace and Stewart, 2007; Piasta et al., 2012). Letter knowledge was generally identified as one of the strongest predictors of later reading and writing success and functions as an important link between the various emergent literacy components (see Adams, 1990; Aram, 2006; see van Kleeck, 2003, for reviews).

The fact that letter knowledge is increased by explicit instruction to grapheme–phoneme conversion rules is not really surprising. However, it was shown that alphabetic instruction within the context of shared book reading has no larger effect on letter knowledge and literacy development compared to alphabetic instruction alone (e.g., Aram, 2006). Thus, the question remains if shared book reading without alphabetic instruction has any effect on the development of the precursors to literacy, i.e., by implicit learning before the alphabetic phase of literacy development (Frith, 1985; Lachmann and van Leeuwen, 2014).

In a recent study, Sim (2012; Sim and Berthelsen, 2014) compared the effects of shared book reading combined with explicit letter instruction with the effects of a special form of shared book reading, i.e., instructed dialogic reading (Whitehurst et al., 1988), without explicit alphabetic instruction on several measures of letter knowledge and print awareness. The author found equally positive effects for both methods when compared with a control group. How could this be explained?

It has been shown that even without explicit letter instruction young children implicitly become aware of the difference between letters and non-letter configurations (e.g., Bialystok, 1995; Brenneman et al., 1996); 4- to 5-year-old children developed a concept about how readable material looks like, from a more figural level to the consideration of specific word constituents, including the order and the orientation of letters within a word (Lachmann and Geyer, 2003; Levy et al., 2006). Children progressively understand that letters are symbols (Deacon, 2000; Lachmann, 2002), i.e., graphemes, even though they still do not understand the meaning. We may call this letter awareness, or more precisely *grapheme awareness*. We suggest grapheme awareness to be one important prior condition for children’s literacy development.

According to the Functional Coordination Model (Lachmann, 2002; Lachmann and van Leeuwen, 2014), learning to read requires the modification of various pre-existing functions, mainly from the auditory (Serniclaes et al., 2005) and visual domain (Pegado et al., 2011; Fernandes et al., 2014; Lachmann et al., 2014), before these become coordinated and automatized in a cross-modal fashion (Blomert, 2011) optimal for reading and writing. This includes the modification of visual strategies originally applied for object recognition (such as pictures in book). These are predominantly holistic (both holistic and analytic processing is possible, holistic processing is, however, faster and less effortful in object recognition, see Lachmann and van Leeuwen for discussion). The learning of grapheme–phoneme conversion rules in the alphabetic phase (Frith, 1985), however, requires analytic strategies (Frith, 1985; Lachmann and van Leeuwen, 2014; Lachmann et al., 2014), including view-point dependency and symmetry-/context suppression (Lachmann, 2002; Lachmann and van Leeuwen, 2007; Pegado et al., 2011; Fernandes et al., 2014). Thus, grapheme awareness may be seen as a very first step in this process of reading-specific modification of visual strategies required for learning to read and write.

Accordingly, we argue that shared book reading supports the development of important prior conditions for learning to read and to write (Goodman, 1986; Robins et al., 2012; Lachmann and van Leeuwen, 2014; Sim and Berthelsen, 2014), even before or at a very early stage of formal reading instruction, i.e., before children learn grapheme–phoneme correspondences (i.e., in the logographic phase of learning to read; Frith, 1985). Therefore, in the present study, we investigated the effects of shared book reading on *grapheme recognition*, i.e., we did not test the effect on letter naming, as was done in all studies in which shared book reading was combined with any form of explicit letter instruction (e.g., Piasta et al., 2012), but rather how well a child recognizes a grapheme within a set of non-letter distractors. We consider this performance to be a measure of *grapheme awareness*.

In an eye-movement study Evans and Saint-Aubin (2009) showed, that during shared book reading children mainly look at the pictures on the page and did not fixate very often on the printed text (see also Justice and Lankford, 2002; Evans and Saint-Aubin, 2005; Justice et al., 2005). Therefore, the question still remains whether shared book reading without explicit alphabetic instruction really does have an effect on grapheme awareness, and if so, whether it depends on formal instruction of teachers about communication strategies during shared book reading, or not (Whitehurst et al., 1988). We tested these questions in the present study.

We implemented two methods of shared book reading in the present study. Method 1 – Literacy enrichment: A large number of additional books chosen as optimal for shared book reading was provided in the kindergarten. The children were encouraged to take these books home, and in a letter the parents were requested to take advantage of the additional books and to read them together with their children. We expected an increase of shared book reading activities at home (Robinson et al., 1996; Wade and Moore, 1996) and, as a consequence, an increase of expressive vocabulary (Sénéchal et al., 1996). The main question was, however, if this literacy enrichment would also increase grapheme awareness.

Method 2 – Teacher training: Kindergarten teachers participated in a formal dialogic reading instruction on reading techniques using an established training program (Buschmann and Jooss, 2011) for shared book reading. As for literacy enrichment, we expected a positive effect of teacher training on children’s expressive vocabulary (Hargrave and Sénéchal, 2000). The main question, here too, was, if teacher training has an effect on grapheme awareness.

A further group received both methods: literacy enrichment and teacher training, in combination. The two methods operate in different contexts of shared book reading, home vs. school. All groups were compared with a control group which received neither of the two interventions. These results in a four-group longitudinal design allowing to test which of the two used methods may be more effective and if a combination is the most effective approach for improving vocabulary and/or grapheme awareness.

Quantity and quality of literacy environment and interaction during shared book reading have been found to be strongly correlated with socioeconomic status (SES; Hart and Risley, 1995), in particular with family income and parental education (McCormick and Mason, 1986; Adams, 1990; Payne et al., 1994; Hart and Risley, 1995; Sénéchal et al., 1996; Arterberry et al., 2007). Therefore, in the present study, care was taken to select groups with comparable SES (see the Materials and Methods section for details).

## Materials and Methods

### Participants

In total, 69 children (43 male) from four German kindergartens, all born in Germany, who ranged in age from 3;0 to 4;8 years (*M* = 4.12, *SD* = 0.54) at the time of their initial assessment, participated in the present longitudinal study. In Germany, about one third of the children younger than 3 years and about 90% of the children aged between 3 and 6 (maximum 7) years attend a kindergarten (Statistisches Bundesamt, 2012), the majority of them from morning to late afternoon, including lunch.

The participating four kindergartens each have several kindergarten groups, more or less mixed in age, with several teachers minding a group of 10 to 25 children. These kindergarten groups are not considered as fixed “classes,” however, they were kept constant during the period of data collection. As standard in Germany (Niklas and Schneider, 2013), there was no formal reading and writing instruction provided in any the participating kindergartens.

Four samples of children, one from each of the four participating kindergarten centers, were created in a way that these did not differ statistically in mean age, age-range from 3 to 4 years, gender, mean time spent in kindergarten per week, number of books for children and adults in household (note that in the literacy enrichment group the number of books in household, except children books, even though being about twice as high as in the other groups, did not differ significantly; two parents from this group held an exceeding collection of dime novels), preferred leisure time activities (e.g., TV watching, computer gaming, playing outside), frequency of visiting public libraries (see **Tables 1**, **2** for details), and, most important, in SES (family income, school-leaving qualification and professional situation of the parents; see **Table 2**). These factors were evaluated using a family socio demographic questionnaire (Simon and Sachse, 2013) completed by all parents before the data collection. The majority of the parents in our study had high or medium school level qualification and a family net income of more than 2000 Euros per month (see **Table 2**), which both is comparable to the total population in Germany (59 and 54%, German Federal Statistical Office, 2016).

**Table 1:** Comparisons of the four groups (group 1 = literacy enrichment, group 2 = teacher training, group 3 = combination, group 4 = control group) regarding gender, age, time spent in kindergarten and the number of books in each household.

	Group 1 (*N* = 13)	Group 2 (*N* = 18)	Group 3 (*N* = 17)	Group 4 (*N* = 21)	* F*	* p*
Male	7	13	13	10	0.56	0.64
Female	6	5	4	11	0.17	0.91
	
	***Mean*** **(*SD*)**	***Mean* (*SD*)**	***Mean* (*SD*)**	***Mean* (*SD*)**	***F***	***p***
	
Age in years	4.24 (0.56)	4.08 (0.63)	4.22 (0.42)	4,02 (0.55)	0.66	0.58
Time spent in kindergarten per day in hours	6.69 (0.86)	6.12 (1.41)	6.64 (1.34)	7.00 (1.00)	1.71	0.18
Number of children’s books in household	32.38 (19.17)	23.75 (18.16)	29.85 (25.65)	25.95 (30.17)	0.37	0.78
Number of books in household	145.00 (274.31)	55.71 (88.27)	47.92 (69.46)	45.24 (47.40)	1.49	0.23

*Statistical comparisons were conducted with univariate ANOVAs. The last two columns depict the corresponding *F-* and *p*-values*.

**Table 2:** Comparisons of the four groups regarding preferred leisure time activities, parents’ level of education, professional situation, frequency of public library visits and family net income.

Variable	χ^2^	*p*	Relative frequencies for the whole sample in %			
Preferred leisure time activities:			Each day	Some days a week	Once a week	< Once a week	Never
Watching TV	9.05	0.43	46.97	34.85	15.15	3.03	0
Listening to music/singing	8.85	0.72	37.88	39.39	10.61	7.58	4.54
Books (alone)	10.64	0.56	30.30	46.97	10.61	10.60	1.52
Shared book reading	12.34	0.42	53.03	33.33	7.58	4.54	1.52
Playing outside	13.28	0.15	54.54	34.85	9.09	1.51	0
Playing alone	10.56	0.57	37.88	40.91	9.09	4.54	7.58
Playing with other children	11.93	0.22	63.64	27.27	3.03	6.06	0
Computer games	19.48	0.08	3.08	6.15	4.62	18.46	67.69
Highest school level qualification	8.83	0.46	None 1.56	Low 12.5	Medium 42.19	High 43.75	
Highest professional situation	13.35	0.34	Full-time 78.46	Part-time 12.30	Education 1.54	No job 3.08	Other 4.62
Frequency of public library visits	11.09	0.27	Never 70.77	Rarely 13.85	Sometimes 9.23	Often 6.15	
Net income	21.37	0.44	<1000 € - 8.77	1000–2000 € - 38.60	2000–3000 € - 38.60	3000–4000 € - 12.28	>4000 € - 1.75

*Relative frequencies for the whole sample are provided for each category. Statistical comparisons were conducted with χ^*2*^ tests. The first two columns depict the corresponding χ^2^ and *p*-values.*

### Procedure

The authors received Institutional Review Board Approval for this study. The study was carried out in accordance with the recommendations of The German Society of Psychology, with written informed consent from the parents of all children. All parents gave written informed consent in accordance with the Declaration of Helsinki. The protocol was approved by the administration of the City of Kaiserslautern, Council for Youth and Sports. Each of the four samples was randomly assigned to serve as one of the three shared book reading intervention groups (experimental groups): (1) Method 1 = literacy enrichment group (*N* = 13); (2) Method 2 = teacher training group (*N* = 18); (3) combination group: Method 1 and 2 combined (*N* = 17); or as the control group: none of the methods were applied until post-test (*N* = 21). See **Table 1** for sample description.

The literary enrichment group and the combination group, each acquired 100 additional different storybooks in order to establish a library system. The storybooks were chosen according to their potential to support vocabulary growth (following guidelines and suggestions by stiftunglesen.de; kinderbuch-couch.de; kidsbestbooks.com; lesestart.de) with colorful pictures and related text on each single page, in order to support emergent literacy. Each of these storybooks was registered with a code and positioned within easy reach of the children. A letter was sent to all parents in which they were informed about the availability of these books and encouraged to read them with their children at home “as often as possible.” Managed and controlled by a graduate student, once a week the children could borrow a new book after bringing back the one borrowed the week before. This procedure was chosen in order to acquaint the children with library rules. There was, however, no financial consequence or any kind of punishment if the book was lost or damaged; borrowing a new book was still possible. Children were free to choose any storybook; the graduate student did not pressure nor make any suggestions. In order to inform the parents, each child received a form with their name and the date when the book was borrowed and when it should be returned.

For teacher training (Method 2), formal instruction was given to the teachers of the two groups for which this method was applied, the teacher training group and the combined group, using one component of the *Heidelberger Interaktionstraining für pädagogisches Fachpersonal zur Förderung ein- und mehrsprachiger Kinder* – HIT (Buschmann and Jooss, 2011), namely shared book reading. The goal of the training, which integrates dialogic reading techniques, is to directly increase the opportunities for children’s language development through shared book reading. Dialogic reading is an intervention approach, originally developed by Whitehurst et al. (1988), that involves high levels of adult–child language interactions during book reading. The program follows three main principles: (a) encouragement of the child to participate, (b) provision of feedback to the child, and (c) adaptation of the adult’s reading style to the child’s language ability. The child is supposed to be active while the adult is reading; the child learns to become a storyteller.

An important part of the teacher training program is that teachers learn to select the right storybook for optimal shared book reading activities. The storybooks should, according to the authors, contain colorful illustrations with related text at each single page. The illustrations should motivate to interactive communication. In order to foster this, the topic of the storybook should meet the interest of the child and the text should include terms and concepts appropriate to the level of her/his cognitive development.

Sixteen teachers completed the teacher training (teacher training group, *N* = 7; combined group, *N* = 9). The training was conducted by a professional instructor, one of the authors of HIT (Buschmann and Jooss, 2011), for a total charge of €3,900 Euros. In order to control for the quantity and the quality of the instruction between the groups, the teachers of both groups were randomly assigned to two mixed training classes. The shared book reading training was conducted in three sessions of four 1-h lessons each with an interval of 1 month between each session. At the end of each session, the teachers received written material (about 10 pages including pictures) in an understandable and motivating form with a synopsis of the strategies learned at the sessions (cf. Buschmann and Jooss, 2011). The training sessions were conducted in a seminar room at the University of Kaiserslautern. The training contained verbal instructions, video demonstrations, model learning, and joint elaboration of activities. Additionally, at the end of the final session coaching was offered, i.e., before session 3 the participants were asked to apply what they had learned in practice and to videotape their shared book reading activities for professional supervision and feedback. Four digital video cameras (two per group) were provided for this purpose.

The teachers were instructed to apply the dialogic book reading strategies three to four times a week in a 1:1 manner, i.e., with one child at time, for approximately 15 min per child, in a quiet room. Each teacher was in charge of three to four children. The teachers used storybooks available in the classroom book corners, if they were in accordance with what they have learned in the training about the selection of storybooks (see above). This holds also for the combination group, i.e., the books provided additionally to this group (Method 1) were not used for Method 2.

In reading logs, for every day (Monday–Friday), each teacher marked her/his frequency of shared book reading activities with each of the three to four children she/he was assigned to. Both kindergartens were visited weekly to collect the logs, to check compliance and to provide guidance.

The control group did not participate in any special instruction or activity. At the end of the study this kindergarten received €300 worth of books as a thank-you gift.

### Measures

In order to measure expressive vocabulary in preschool age before and after shared book reading intervention, a subtest of the Revised Vocabulary Test for 3- to 5-year-old Children (“*Aktiver Wortschatztest für 3- bis 5-jährige Kinder* – AWST-R 3-5”; Kiese-Himmel, 2005) was used. Children were instructed to look at a picture and to verbalize what they saw and what action could be described by asking them “what is it” (“Was ist das?”) – for vocalization of substantives, or “what are they / is she / he doing” (“*Was macht sie/er…?”*) for verbalization of verbs. According to the tests manual, one point was given for each correct answer. The total of 74 items was divided in two parallel forms of 37 items each. The two forms had equal difficulty. One version was used for pre-testing and the other for post-testing. The pictures were presented on the screen of a laptop computer.

In order to measure grapheme awareness, we developed a task which tested children’s familiarity with grapheme forms. In each of 10 trials they had to identify a grapheme presented amongst three non-letter distractors displayed simultaneously on the computer screen, by pointing to it without having to name it. Thus, the test could be applied before formal instruction on grapheme–phoneme conversion rules. For each of these 10 trials a display with four items was presented individually. Each display consisted of one upper case letter and three distractors: one pseudo-letter, one number, and one symbol, each presented in varying location either in the upper left/right, lower left/right part of the screen. All distractors looked similar to letters and may even be used in text (symbols and numbers). Thus, the task required more than just an idea how readable material looks like (Bialystok, 1995), it rather requires familiarity with concrete forms of a real graphemes. The letter items were D, M, W, P, G, H, S, K, F, R, deformed versions of them served as pseudo-letters. Further ten symbols from regular keyboards (e.g., #, $, %) and the ten first numbers (e.g., 1,2,3) were used as distractors.

The question given orally to the children after the onset of each trial was “where is the letter” (“*Wo ist der Buchstabe?”*). For each correct answer, the child received one point. The chance level was 25% in total. Considering a 95% confidence interval for chance level a score up to 4 must be considered as within chance level. Four children in the combination and the control group, respectively, and two children in the literacy enrichment and teacher training group, respectively, reached a score higher than this. These frequencies do not differ between the groups, χ^2^(3) = 1.02 (*p* = 0.80).

The duration of conducting both tests together (first expressive vocabulary, measured with a subtest of the AWST-R 3–5, followed by the grapheme awareness task) did not exceed 30 min. After 6 months intervention time, these two tests, were repeated in the posttest section.

## Results

### Compliance

We registered parameters of compliance in order to test whether the effects of both methods is influenced by the frequency of which children were exposed to shared book reading. Literacy enrichment group and the combination group were comparable regarding the number of borrowed books, frequency and length of shared book reading with a parent, number of books forgotten to return, and frequency of children’s absence from kindergarten (see **Table 3**). Regarding teacher training group no difference between the teacher training group and the combined group in the frequency of absence of teachers and children was found. The higher frequency of teacher–child shared book reading in the combination group as compared to the teacher training group did not reach significance after Bonferroni correction (see **Table 3**).

**Table 3:** Comparisons of the three intervention groups (group 1 = literacy enrichment, group 2 = teacher training, group 3 = combination group) regarding measures of compliance.

	Group 1 *Mean* (*SD*)	Group 2 *Mean* (*SD*)	Group 3 *Mean* (*SD*)	*t*	*p*
Number of borrowed books	12.54 (4.28)		15.18 (3.74)	1.80	0.08
Frequency of shared book reading with parents	20.15 (11.25)		23.53 (8.46)	0.94	0.36
Number of forgotten books	6.38 (4,48)		5.60 (3.64)	0.51	0.61
Frequency of children’s absence	3.77 (2.09)		2.93 (2.49)	0.95	0.35
Frequency of shared book reading with teacher		16.11 (5.31)	22.06 (10.40)	2.15	0.04
Frequency of teacher’s absence		15.39 (13.31)	12.35 (7.43)	0.83	0.42
Frequency of children’s absence		25.11 (15.50)	17.88 (12.88)	1.50	0.14

*Statistical comparisons were conducted with *t*-tests. The last two columns depict the corresponding *t-* and *p*-values*.

### Influence of the Own First Name on Grapheme Awareness

Knowledge for letters which are part of the child’s first name has been shown to be enhanced in some studies (Treiman and Broderick, 1998). To rule out corresponding biasing effects on the grapheme awareness task, χ^2^ tests were performed to check whether the frequency of the target letters of this task is comparable within the prenames of the children for the four groups. The occurrence of the target letters within the prenames was comparable in all four groups, χ^2^(3) = 7.00 (*p* = 0.86). Moreover, the occurrence of the target letters as the first letter of the prename was comparable in all four groups, χ^2^(3) = 4.09 (*p* = 0.25). There were no correlations between the number of target letters within the own prename and the performance in the grapheme awareness pretest, *r* < 0.01 (*p* > 0.99), and posttest, *r* = -0.03 (*p* = 0.83), indicating that the results of the grapheme awareness task were not influenced by the occurrence of the target letters in the children’s first names.

### Treatment Effects

AWST-R scores (expressive vocabulary as measured by the number of correct words in the AWST-R 3-5) and grapheme awareness scores (number of correct letters in the letter recognition task) were analyzed including the factors Time (before vs. after the intervention) as within-participants factor, and Group (Library Group, Teacher Training Group, Combination Group, Control Group) as between-participants factor. Since these dependent variables were associated (AWST-R Pretest with grapheme awareness Posttest: *r* = 0.32, *p* < 0.01; AWST-R Posttest with grapheme awareness Posttest: *r* = 0.29, *p* = 0.02) a multivariate analysis of variances (MANOVA) was run first, followed by univariate ANOVAS of each dependent variable. Partial eta squared and Cohen’s *dz* (Cohen, 1988) are reported as measures of effect size.

The Box’s *M* test revealed homogeneity of the covariance matrices, χ^2^(30) = 20.33 (*p* = 0.91). In line with this finding, the Levene test revealed homogeneity of variances between groups for both, the AWST-R, *F*(3,65) = 0.30 (*p* = 0.83), and the grapheme awareness task, *F*(3,65) = 0.30 (*p* = 0.82).

Since Kolmogorov–Smirnov tests revealed that grapheme awareness scores were not normally distributed (*p* < 0.01, for the data at both time points), for *post hoc* analyses of this dependent variable we additionally used bootstrapped *t*-tests, appropriate for data not normally distributed (Bollen and Stine, 1990; Preacher and Hayes, 2004).

The MANOVA revealed a main effect of Group, Wilk’s λ = 0.76 (*p* < 0.01, $\eta_{p}^{2}$ = 0.13), a main effect of Time, Wilk’s λ = 0.46 (*p* < 0.01, $\eta_{p}^{2}$ = 0.54), and an interaction between Time and Group, Wilk’s λ = 0.78 (*p* = 0.01, $\eta_{p}^{2}$ = 0.12). Significant improvements over time were found for all groups, all *ts* < -2.75 (*ps* < 0.01), with the exception of the control group, *t*(20) = -0.93 (*p* = 0.35).

**FIGURE 1:**
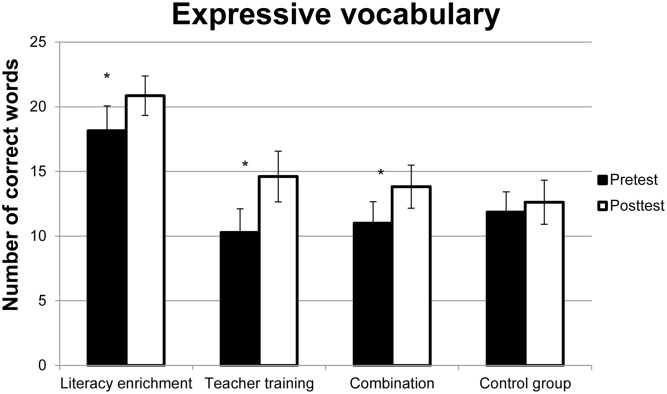
**Mean performance for the four groups before and after the interventions in the expressive vocabulary task**. Error bars indicate standard error of the mean. Asterisks mark significant improvements over time.

The ANOVA on the AWST-R scores revealed a main effect of Time, *F*(1,65) = 30.66, *p* ≤ 0.01, $\eta_{p}^{2}$ = 0.32, with a higher performance for the second compared to the first time point, *t*(68) = -5.54, *p* ≤ 0.01. Moreover, there was a main effect of Group, *F*(3,65) = 3.65, *p* = 0.02, $\eta_{p}^{2}$ = 0.14. The Library Group achieved higher scores compared to all other groups, all *ts* ≥ 2.74, *p* ≤ 0.01, whereas no differences were found between the remaining three groups, all *ts* ≤ 0.09. There was an interaction between Time and Group, *F*(3,65) = 2,73, *p* ≤ 0.05, $\eta_{p}^{2}$ = 0.11. A significant improvement over time was found for all intervention groups, all *ts* ≥-2.48, *ps* ≤ 0.016 (Library Group: *dz* = 0.75; Teacher Training Group: *dz* = 1.38; Combination Group: *dz* = 0.63). No difference over time was found for the Control Group (see **Figure 1**).

**FIGURE 2:**
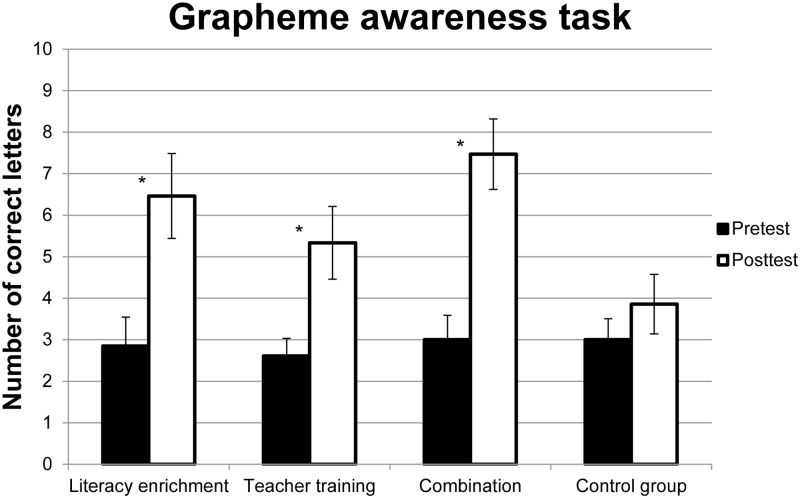
**Mean performance for the four groups before and after the interventions in the grapheme awareness task**. Error bars indicate standard error of the mean. Asterisks mark significant improvements over time.

For the second dependent variable, scores in the grapheme awareness task, ANOVA revealed a main effect of Time, *F*(1,65) = 32.26, *p* ≤ 0.01, $\eta_{p}^{2}$ = 0.33, with a higher performance for the second compared to the first time point, *t*(65) = -5.68, *p* ≤ 0.01. For the main effect of Group, *F*(3,65) = 2.60, *p* = 0.06, $\eta_{p}^{2}$ = 0.11, and the interaction between Time and Group, *F*(3,65) = 2.55, *p* = 0.06, $\eta_{p}^{2}$ = 0.11, there was a clear tendency (6% level), i.e., statistical significance at the 5% Level was narrowly missed. The scores of grapheme awareness were, as mentioned before, not normally distributed. Therefore, additional bootstrapped confidence intervals were conducted (Bollen and Stine, 1990; Preacher and Hayes, 2004). These analyses revealed that all groups, with the exception of the Control Group, improved significantly over time (see **Figure 2**). For detailed results of the bootstrapped *t*-tests, see **Table 4**.

**Table 4:** Improvement over time for each group in the grapheme awareness task.

Group	Mean	Standard error	*p*	Low border of CI	High border of CI	*dz*
Literacy enrichment	-3.62	1.07	0.02	-5.54	-1.69	0.89
Teacher training	-2.72	1.02	0.01	-4.56	-0.83	0.62
Combination	-4.47	1.02	<0.01	-6.29	-2.56	1.04
Control	-0.86	0.86	0.34	-2.57	0.86	–

*Statistical comparisons were conducted with bootstrapped *t*-tests for dependent samples, as the grapheme awareness data was not normally distributed. Confidence intervals (CI) which do not include 0 indicate significant results. The last column depicts the corresponding Cohen’s *dz* values*.

## Discussion

In the present longitudinal study, we investigated the effects of two methods of shared book reading with kindergarten children on the development of expressive vocabulary and grapheme awareness. The latter is assumed to be a precursor of later literacy development. Method 1 was literacy enrichment: an extra library consisting of storybooks especially suitable for the purpose of shared book reading with parents was made available in two kindergartens (literacy enrichment group and combination group), allowing children to borrow one of them weekly. Method 2 was teacher training: teachers participated in a training of shared book reading. The techniques learned in the training should be applied in two kindergarten samples (teacher training group and combination group). While in one kindergarten sample both methods were combined (combination group), in two other kindergartens samples only one of these methods was applied, either literacy enrichment or teacher training, respectively. A fourth kindergarten sample served as a control group.

We expected effects of both methods on expressive vocabulary, as these have been shown in a number of earlier studies (Whitehurst et al., 1994; Wade and Moore, 1996; Wasik and Bond, 2001). Of particular interest, however, was the question of whether grapheme awareness (measured by the ability to identify per trial a letter out of a series of items of which three were non-letter distractors) is also promoted by shared book reading in terms of literacy enrichment and/or teacher training, even without formal alphabetic instruction. This, to the best of our knowledge, has not been investigated before. Yet another question was what happens when both methods are combined.

Overall, results show that both methods of shared book reading affected children’s expressive vocabulary development as well as their grapheme awareness positively. There is no clear evidence that the combination of the two methods had a greater effect on expressive vocabulary and grapheme awareness as compared to when only one method was applied. The results will be discussed in greater detail, separately for expressive vocabulary and grapheme awareness, in the following.

### Shared Book Reading and Expressive Vocabulary

Results from the literacy enrichment group show that simply providing a large number of adequate books to children, followed by an informal encouragement of the parents to read these books with the children at home, improves children’s language development in terms of expressive vocabulary. It seems that the availability of appropriate books in an educational institution connects home and school activities in favor of language development by stimulating literacy activities at home (Wade and Moore, 1996; Deunk et al., 2013): supporting children to experience of the “intimacy” of shared reading in home settings (Merchant, 2008) and making “recreational reading” part of the routine (Morrow and Weinstein, 1986). In fact, many parents expressed their gratitude for the opportunity for their children to borrow good books. Some, for instance, created a special bag for the home–school book transport. It seems that this method simply encourages families’ participation in shared book reading activities (Meyer et al., 2015).

This “opportunity invites” principle seems also to hold true for the children themselves, who were highly motivated to choose a new book each week. Some of them were talking about their expectations regarding the borrowed book or, when returning the book, they judged it, discussed the content and told us whether or not they liked it. Borrowing books may have attracted and increased children’s general attention to books. Such effects have been reported in the literature (Robinson et al., 1996). Moreover, with the frequent use of the borrowing method, children may have acquired skills in how to deal with the wide variety of books, as usually found in public libraries, and have learned how to choose a book that meets their subject expectations (Hurrelmann et al., 1993; Deunk et al., 2013).

The text and pictures of the storybooks used in the present study were optimal for retaining children’s attention and for stimulating language development (stiftunglesen.de; kinderbuch-couch.de; kidsbestbooks.com; lesestart.de), with colorful pictures and related text on each single page, whereas many books which children usually have at home are not of the same standard (Elliott and Hewison, 1994; Marsh, 2003). Thus, literacy enrichment does not only increase the quantity of shared book reading activities but also its quality.

Altogether, we can conclude that literacy enrichment is an effective method which can, due to relatively low costs and without financial liability for the parents, easily be implemented in kindergarten centers. Only one single book out of the 200 we sponsored for literacy enrichment was considerably damaged and only five books were not returned. At the end of the study, the remaining books were still available to all children of the two kindergarten groups.

Results from the teacher training group show that providing formal shared book reading instruction (Method 2) results in positive effects on children’s expressive vocabulary, corroborating the findings of other studies which found similar results of different formal teacher training instruction (Valdez-Menchaca and Whitehurst, 1992; Wasik and Bond, 2001; Buschmann and Jooss, 2011; Simon and Sachse, 2013; Milburn et al., 2014). As a consequence of the training, teachers learned the importance of open questions, how to do corrective feedback, how to use expansions and how to motivate children to speak and to be more active during telling the story. This seems to increase both quantity and quality of shared book reading activities (Whitehurst et al., 1988) and its subsequent impact on language development.

Moreover, teachers learn how to create a scenario for incidental vocabulary learning, which means the development of word knowledge may occur through natural contexts that differ from formal teaching (Valdez-Menchaca and Whitehurst, 1988). In such incidental context through shared book reading, children are exposed to an unstructured rich meaningful environment that can be accessed via language, and the process of acquiring some knowledge is based on children’s spontaneous interest, and not followed by a preselected sequence of topics (Valdez-Menchaca and Whitehurst, 1988). After the child has expressed interest in some subject or aspect of the story (it could be verbally or non-verbally), the teacher can interact by requiring and helping the child to verbalize their thoughts in the context of the story.

Results of the combination group showed that combining the two methods of shared book reading results in positive effects on children’s expressive vocabulary. This matches finding by Mason et al. (1990) and Whitehurst et al. (1994) which showed similar effects of combining shared book reading at home and at kindergarten in children’s language development.

The effect of combining the two methods was, however, not higher when compared to the effects of the methods applied alone, respectively. This may be considered as somewhat surprising, because in this group both methods were applied to the same extent as in the other two groups, respectively, and children were thus exposed to considerably more frequent shared book activities. It seems that effects of shared book reading reached a ceiling effect, i.e., the effect increased with an increase in quantity (Ehmig and Reuter, 2013), however, only to a certain level, and after threshold, cannot be improved further by quantity.

### Shared Book Reading and Grapheme Awareness

Results from the literacy enrichment group show positive effects on grapheme awareness. In earlier studies, effects of various shared book reading activities on letter knowledge were shown (Ezell and Justice, 2000; Aram, 2006; Piasta et al., 2012). In these studies, however, explicit letter instruction was given. Children were, for instance, asked to verbalize the letter names or to point to the requested letter (Sénéchal et al., 1998; Ezell and Justice, 2000). The present results show that even without explicit letter instruction, shared book reading may implicitly help children to become aware of the difference between printed letters and other visual configurations (Goodman, 1986; Robins et al., 2012). This awareness was measured by the ability to identify a grapheme within a set of items including three non-letter distractors, which all looked similar to letters and may even be used in text. Thus, the task requires more than just an “idea how readable material looks like” (Bialystok, 1995), it rather requires familiarity with definite forms of existing graphemes (including their orientation and detailed features), i.e., the awareness that the chosen item represents a real grapheme. We termed this awareness “grapheme awareness.”

The finding that shared book reading increases grapheme awareness is of practical importance because, for German kindergarten children formal letter instruction is not part of the pre-school curriculum. Many children enter Grade 1 (after reaching the age of six) with only little knowledge about letters and without reading ability (Mann and Wimmer, 2002; Niklas et al., 2011). In contrast, US kindergartens, for instance, usually offer activities that encourage learning the letter names and their sounds with activities that require giving pre-reading attention to the phonological segments of words (Mann and Wimmer, 2002). Such cultural differences were also reported for home literacy activities (see Lego Learning Institute, 2003, for an overview). Literacy enrichment can easily be implemented and may help German kindergarten children to be better prepared for learning to read and write in school.

Whereas the effects of literacy enrichment on expressive vocabulary has been discussed in the literature extensively, effects on grapheme awareness were not addressed so far. In the following, we will discuss how the positive effects we have found could be explained.

We observed that when children borrowed a book, they usually first checked its content before making a decision in favor of one. Related activities, such as choosing some books from the bookshelf, exploring and handling them, putting the chosen one in and out of the satchel, etc., provide additional opportunities for contact with written text.

The books from the literacy enrichment method were managed by an external person (research assistant from our lab). In principle, however, this could also be managed by the teachers without taking away too much time from the other activities (Morrow and Weinstein, 1986; Robinson et al., 1996). In fact, when teachers organize the borrowing, it is already part of reading related interaction; teachers can help children to choose a book by talking about the title or the expected content (Deunk et al., 2013), and in the end it is more likely that the child will like the book.

Results from the teacher training group showed a positive effect of the training program on children’s grapheme awareness, even though the program is focused on strategies of expressive language development. It seems, children have still been supported to informally learn how print looks and thus to acquire familiarity with the visual characteristics of the letters and their orientation (Lachmann and Geyer, 2003; Robins and Treiman, 2009; Neumann et al., 2013; Both-de Vries and Bus, 2014).

Results of the combination group showed that combining the two methods of shared book reading results in positive effects on grapheme awareness. Even though the effect size for this group was considerable larger than for the groups in which the methods were applied alone, there was no statistical evidence for a difference. Therefore, the finding is identical to what was found for expressive vocabulary. Here too, this may be considered as surprising, because in the combination group both methods were applied to the same extent then in the other two experimental groups, respectively, and children were thus exposed to considerably more frequent shared book activities. As discussed for expressive vocabulary, here too, it could be that the effect of shared book reading on grapheme awareness increases with *quantity*, but only up to a certain level.

We conclude that both methods, literacy enrichment and teacher training trigger experiences with print through shared book reading even without formal letter instruction (Sonnenschein and Munsterman, 2002; Sawyer et al., 2014). Thereby children begin to distinguish letters from other visual configurations and, consequently, will learn that letters are symbols representing language elements and to visually perceive and process graphemes analytically. This modification of visual perception is considered to be an important precondition of literacy development, in particular to learn grapheme–phoneme correspondences (Lachmann, 2002; Lachmann et al., 2012; Lachmann and van Leeuwen, 2014). This is not restricted to transparent orthographies, such as German (Ehri, 1998; Lachmann et al., 2010).

To the best of our knowledge the present study is the first one in which the effect of shared book activities without letter instruction on grapheme awareness is measured. For gaining letter knowledge, i.e., for learning the grapheme-to-phoneme conversion rules in the alphabetic phase of literacy acquisition (Frith, 1985), the children have to process graphemes differently than similar non-letter configurations (Lachmann and van Leeuwen, 2014). Grapheme awareness may thus be considered as an important early precondition which promotes the modification of visual processing of graphemes toward the analytic processing required for letter knowledge. Since, in turn, early letter knowledge has been shown to be one of the strongest predictors of later reading and writing success (Schatschneider et al., 2004; Leppänen et al., 2008), grapheme awareness may be considered an important first step in the process of learning to read and write (Lachmann, 2002; Lachmann and van Leeuwen, 2014; Lachmann et al., 2014).

## Limitations

Although the present study has reached its aim, there are some limitations to be mentioned. A total of 69 children participated in the present study. At group level the sample size is quite small. This works against our hypotheses. Statistical tests were carefully chosen, taking into account the sample size, the associations between dependent variables and parameters of distributions. Moreover, every endeavor has been made to control for factors such as gender, age, SES, and others. Therefore, the present results are interpretable. Nevertheless, the sample size is not large enough for a generalization of the present results, and the study should, therefore, be considered as pilot study, the first one on the effects of shared book reading without additional letter instruction on grapheme awareness, motivating new research.

There are some limitations related to both methods applied, literacy enrichment and teacher training. Regarding literacy enrichment, the frequency of shared book reading at home was recorded by a graduate student by simply asking the children how often they have read the book at home with the parents. Future studies should control for this variable by sending reading logs to the parents for a clear registration of how often the books were read during the week.

Regarding Method 2, the teacher training included individual or small group coaching by supervisors on the basis of videotaped interactions between teacher and children during shared book reading activities in kindergarten. None of the teachers, however, took advantage of this offer. Therefore, no coaching was performed at all. This may have restricted the effectiveness of the training (Neuman and Cunningham, 2009; Powell et al., 2010; Dickinson et al., 2014). Furthermore, it was not controlled if the teachers really followed the instructions given in the training for the choice of books. What books were finally chosen, however, may also have influenced the quality of shared book reading (de Jong and Bus, 2002; Kaderavek and Justice, 2005; Moschovaki and Meadows, 2005; Dickinson et al., 2014), and thus, should be controlled in future research. The children should be involved in the process of choosing the books. This would lead to an additional exposure to print and may better meet the interests of the children (comparable to literacy enrichment method, see above). A last problem with the teacher training to be mentioned is that teachers did not always follow the instruction to perform shared book reading strictly in a one-to-one manner.

## Conclusion

In the present study, we investigated effects of shared book reading on the development of expressive vocabulary and grapheme awareness without letter instruction in German kindergarten children. Expressive vocabulary was measured with a standardized German vocabulary test (AWST); grapheme awareness was measured by a task requiring children to identify graphemes presented amongst non-letter distractors.

Two methods for promoting shared book reading were investigated, literacy enrichment by providing additional books, and teacher training in shared book reading strategies consisting of adopted components of the *Heidelberger Interaktionstraining für pädagogisches Fachpersonal zur Förderung ein- und mehrsprachiger Kinder* – HIT.

Both of these methods resulted in positive effects on children’s expressive vocabulary. This finding is in line with earlier studies (Valdez-Menchaca and Whitehurst, 1992; Wasik and Bond, 2001; Buschmann and Jooss, 2011; Simon and Sachse, 2013; Milburn et al., 2014) and shows that improving the language interaction by a specific training of teachers and even a simple literacy enrichment in kindergarten may increase the quantity and quality of shared book reading activities and finally promote the language development of the children.

Both methods also had positive effects on grapheme awareness. This is the first study showing that even though no formal letter instruction was given. The children became more familiar with graphemes, i.e., with letter forms and orientations. Even though there is no direct evidence it could be concluded that they became aware of the fact that, in contrast to other visual configurations, graphemes are abstract representations of elements of spoken language (without knowing what in particular).

We consider grapheme awareness to be a first step in the process of reading-specific modification of predominantly holistic visual strategies toward an analytic processing of graphemes (Lachmann, 2002; Lachmann and van Leeuwen, 2014), which is required for learning grapheme–phoneme conversion rules in the alphabetic phase (Frith, 1985) of reading acquisition. Therefore, we should consider shared book reading not only to be a tool for promoting the development of expressive language, but also for supporting the process of learning to read.

## Author Contributions

TL is the initiator and supervisor of the study and co- as well as senior author. PW was conducting the study and is first author. CC contributed in running the analyses and is shared first author.

## Conflict of Interest Statement

The authors declare that the research was conducted in the absence of any commercial or financial relationships that could be construed as a potential conflict of interest.
